# Supplementary material for: Bimodal nonlinear dendrites in PV+ basket cells drive distinct memory-related oscillations
Source: iScience. 2025 Oct 4;28(11):113699. doi: 10.1016/j.isci.2025.113699 (PMC12590550; doi:10.1016/j.isci.2025.113699)
Supplement: Document S1. Figures S1–S6, Tables S1–S5, and Data S1 [file mmc1.pdf]

iScience, Volume *28*

Supplemental information

Bimodal nonlinear dendrites in PV+ basket cells drive distinct memory-related oscillations

Alexandra Tzilivaki, Matthew Evan Larkum, and Dietmar Schmitz

Supplementary Information

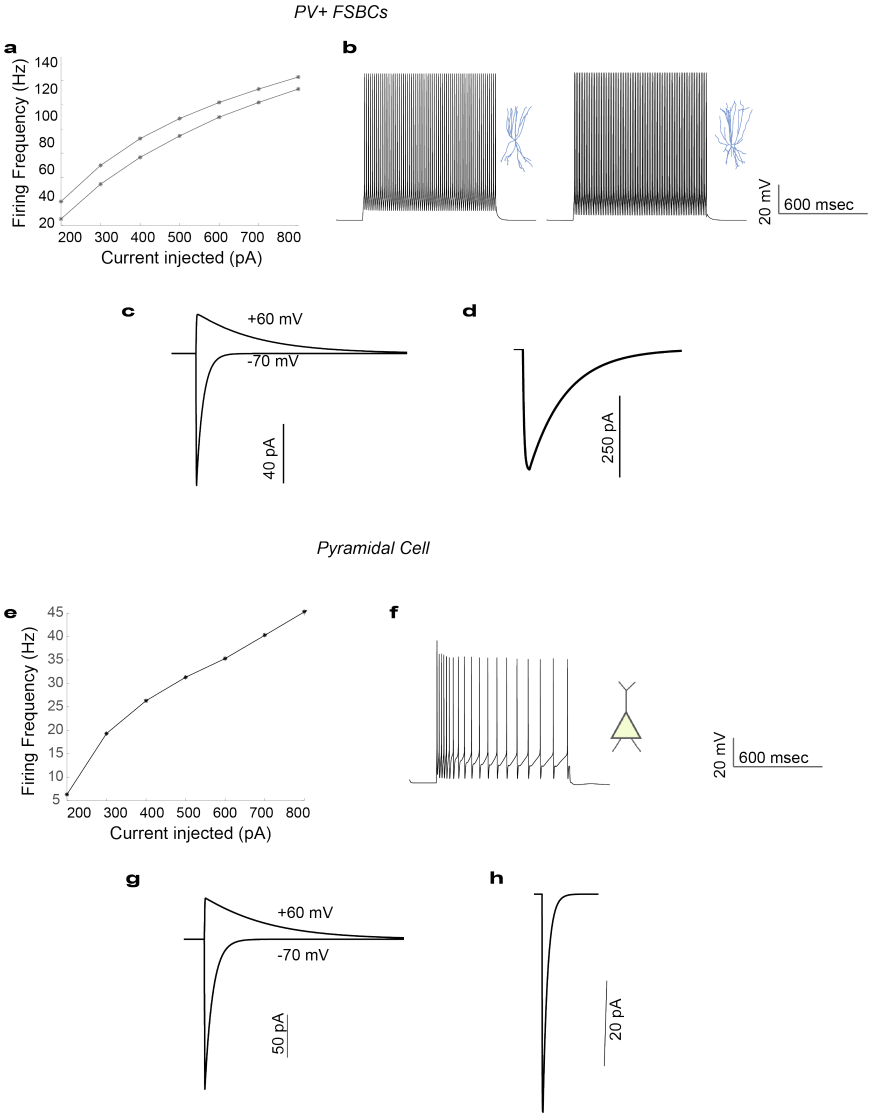

***Figure S1. Electrophysiological calibration and responses of the models. a-c. PV+** FS BCs responses. **a.** Frequency- Injected current (FI) curve upon current clamp simulation in the cell bodies of the two multicompartmental PV+ FSBCs (1 sec duration). **b**. Firing profiles of the two FSBCs after depolarizing current injections in the cell bodies (300 pA, 1 sec). PV+ FSBCs exhibit the typical high-frequency firing pattern. **c.** CP-AMPA (−70 mV) and NMDA (+60 mV) currents upon stimulation as per [59]. Traces represents the mean of the two multicompartmental FSBCs. **d.** A three-step voltage clamp of voltage changes from −70 mV to 10 mV (duration 1 msec) and back to −70 mV was used to produce inhibitory current. During the validation of this current, the reversal potential of Cl− was adjusted from −80 to −16 mV, in order to reproduce the experimental set up of [62], Mean trace of the two FSBCs responses. **e-h** Pyramidal Cell responses. **e.** FI curve of the Pyramidal Cell model same approach as in a. **f.** Firing profile of the Pyramidal Cell under current injection (300 pA for 1 sec) at the cell body. The model successfully represents the typical experimental phenotype shown in [55]. **g.** AMPA (-70 mV voltage clamp) and NMDA (60 mV voltage clamp) currents of the Pyramidal Cell model mimic the experimental data of [57]. **h.** Inhibitory current calibration based on experimental data from [63].*

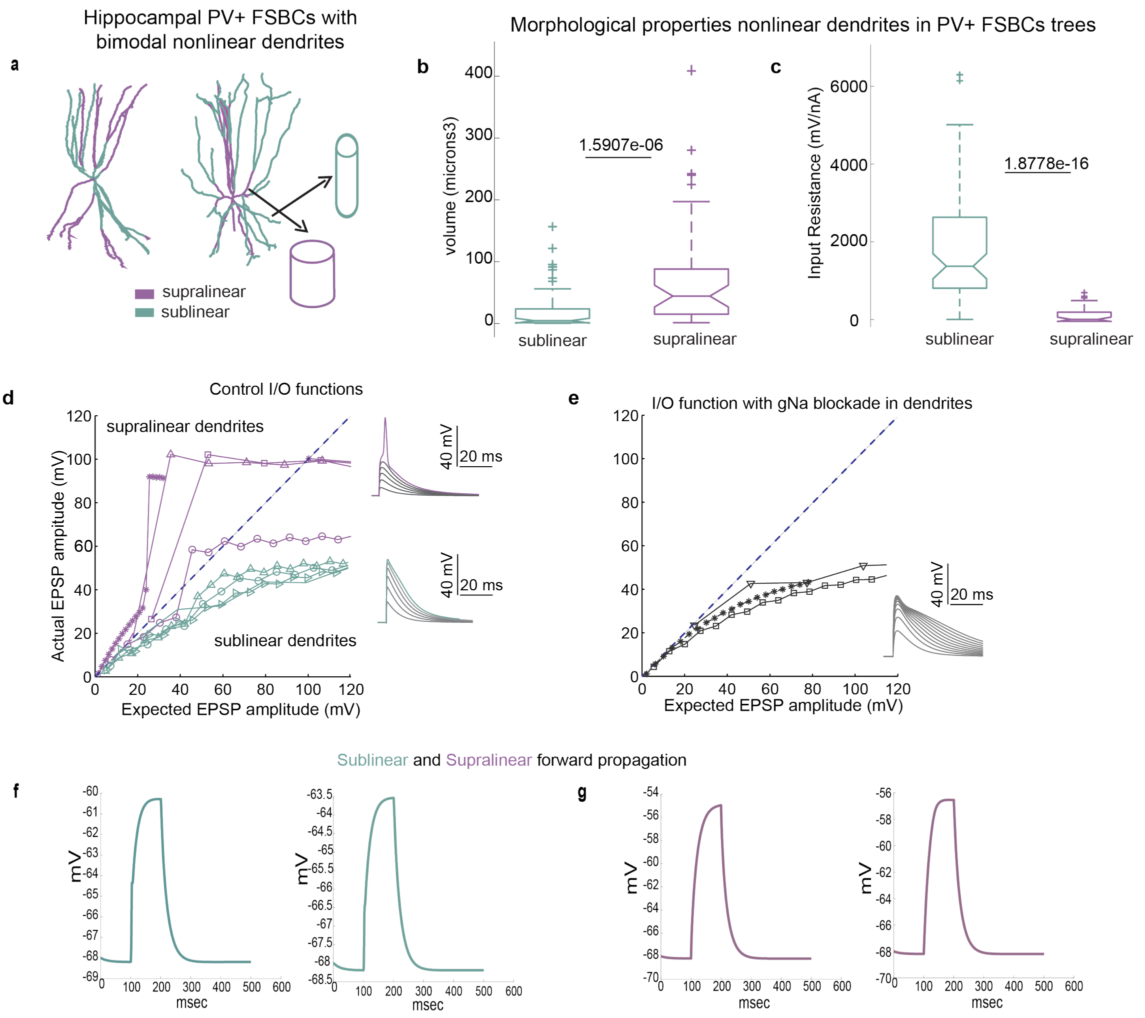

***Figure S2**. **Mechanisms of bimodal nonlinear dendritic integration in Multicompartmental Models of Hippocampal PV+ FSBCs.** **a**.Illustration of the morphological characteristics of supralinear and sublinear dendrites in bimodal PV+ FSBC models. Supralinear dendrites (purple) are larger, whereas sublinear dendrites (blue) are longer and thinner. **b-c**. Discriminative features between dendrite types: supralinear dendrites have larger volumes (b) and lower input resistance (c) compared to sublinear dendrites. Statistical significance was determined using the Mann–Whitney U test. **d-e**. Supralinear dendrites (purple) can generate local sodium-dependent spikes, whereas sublinear dendrites (blue) cannot (d). Blocking active sodium conductance in PV+ FSBCs dendrites completely abolishes the supralinear mode (e). **f-g**. Forward propagation efficiency in bimodal nonlinear dendrites of PV+ FSBCs: Current injection (100 pA) at randomly selected dendrites and recording at the soma show that sublinear branches (f) propagate signals less effectively compared to supralinear branches (g). Panels a,d,e were adopted from [33] Data show mean and std values.*

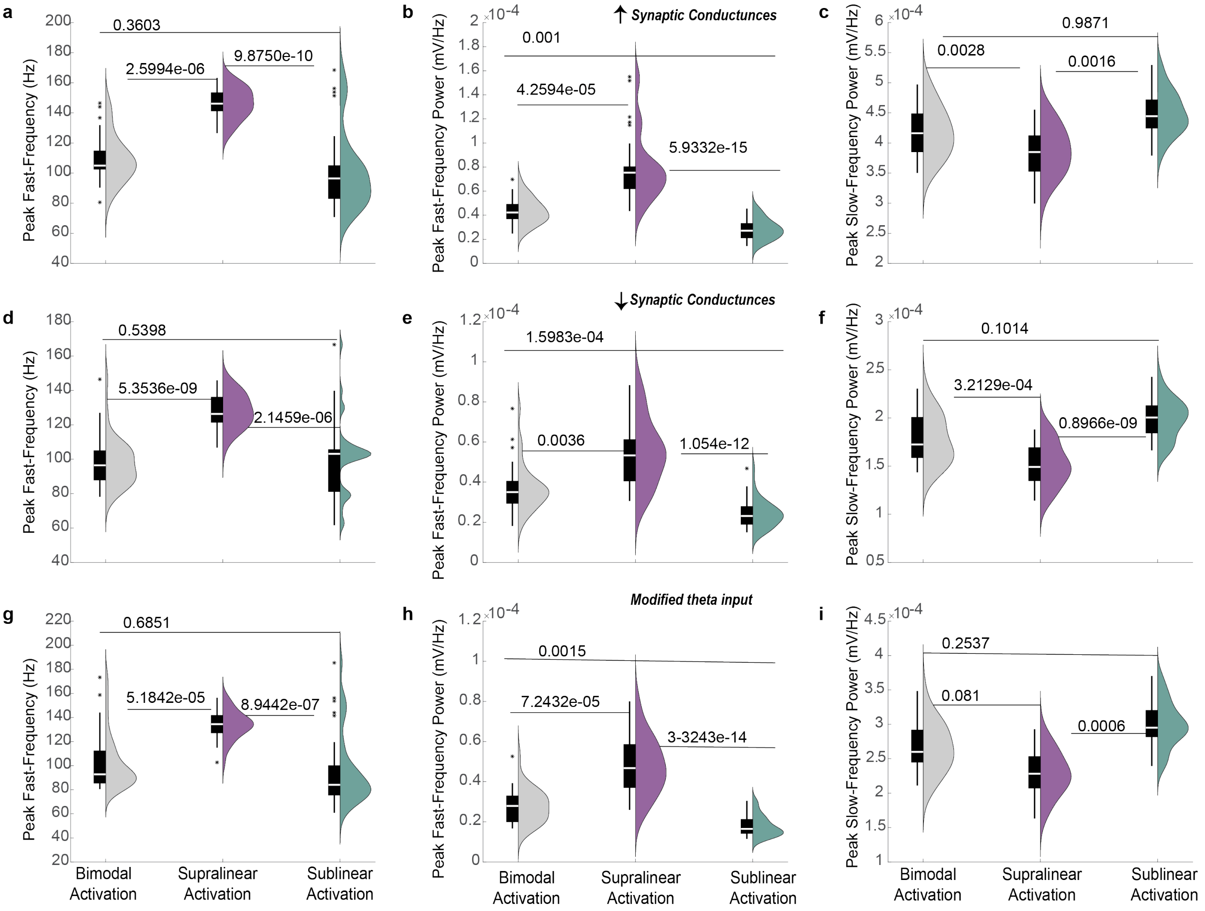

***Figure S3. Robustness/Sensitivity Analysis on Synaptic and Input Parameters. a-c**. 15% increase in the synaptic conductance values (applied to both input and network synapses for/from both PCs and Pv+ FSBCs) do not alter the enhancement of fast peak frequency and power observed with supralinear activation. **d-f**. 15% reduction of the synaptic conductance values (for both input and network synapses involving PCs and PV+ FSBCs) also maintain the observed increase in fast peak frequency and power upon supralinear activation. **g-i**. Modifications to input parameters (input phase shifted 180°, peak frequency 5 Hz) similarly do not affect the enhancement of fast peak frequency and power driven by supralinear activation. Statistical analyses for multigroup comparisons were performed using the Kruskal-Wallis test, followed by a post-hoc correction for multiple comparisons. Data show mean and std values.*

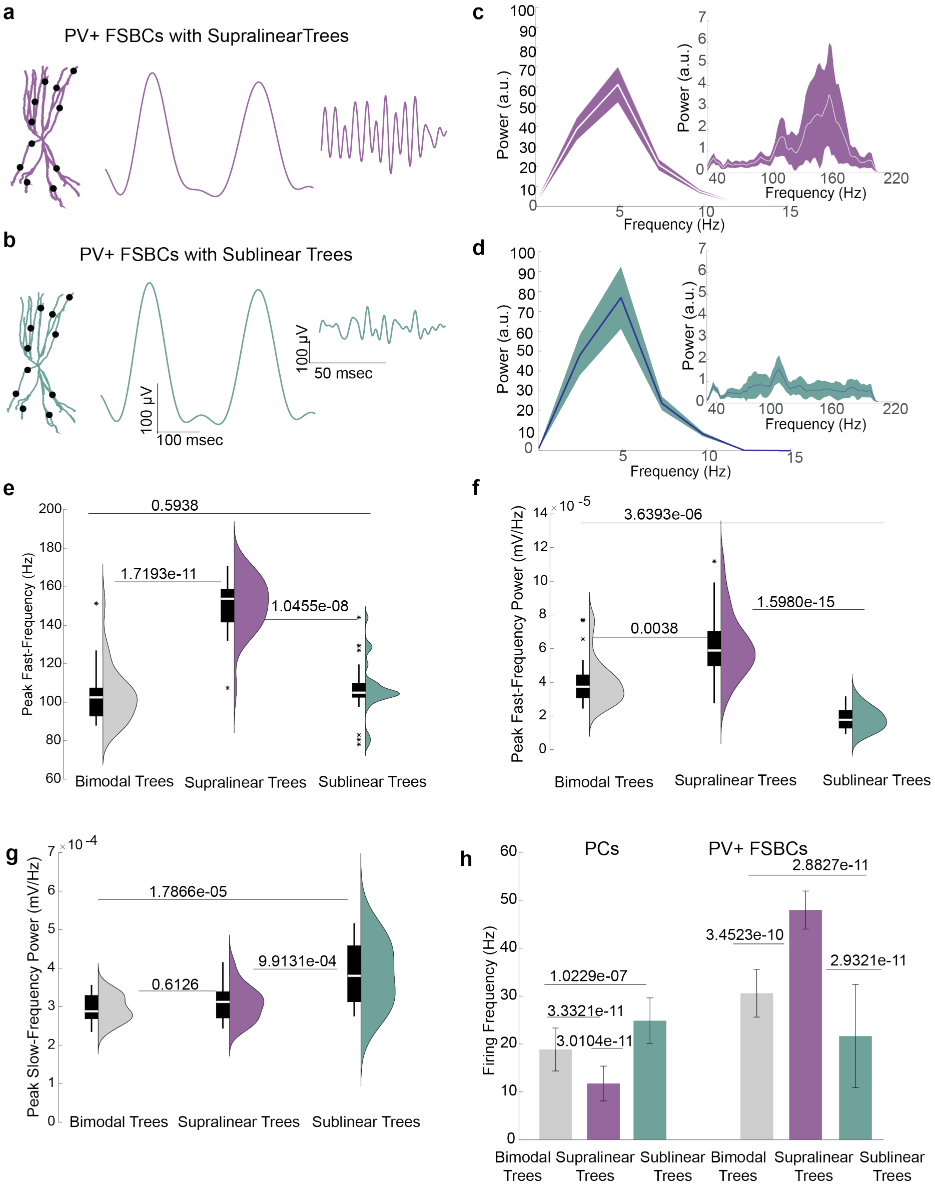

Figure S4. Differential Modulation of Slow and Fast LFP Components by Supralinear and Sublinear Pv+ FSBCs Dendritic Trees. a*. Activation of PV+ FSBCs equipped with purely supralinear dendritic trees, showcasing representative LFP traces bandpassed at slow (3-10 Hz) and high (30-200 Hz) frequencies.* ***b****. Similar to* ***a*** *but displaying activation of PV+ FSBCs with purely sublinear dendritic trees.* ***c-d****. Power Spectrum Density (PSD) plots of the LFP evoked when PV+ FSBCs are equipped with purely supralinear (c) or purely sublinear (d) dendritic trees, highlighting differences in frequency response.* ***e-g****. Comparative analysis of the peak fast-frequency (e) and peak power of fast (f) and slow (g) oscillations for PV+ FSBCs with bimodal (grey), supralinear (purple), or sublinear (blue) dendritic trees. Data are derived from 30 random simulation trials.* ***h****. Firing activity of the PCs and PV+ FSBCs populations within the microcircuit network across 30 random simulation trials. Activation of supralinear PV+ FSBC dendritic trees results in a decreased E/I balance compared to both bimodal (control) and sublinear trees. Statistical analyses for multigroup comparisons were conducted using the Kruskal-Wallis test followed by a post-hoc correction for multiple comparisons. Paired comparisons and p-values were calculated using the Mann-Whitney U test for data with unequal variance. Data show mean and std values.*

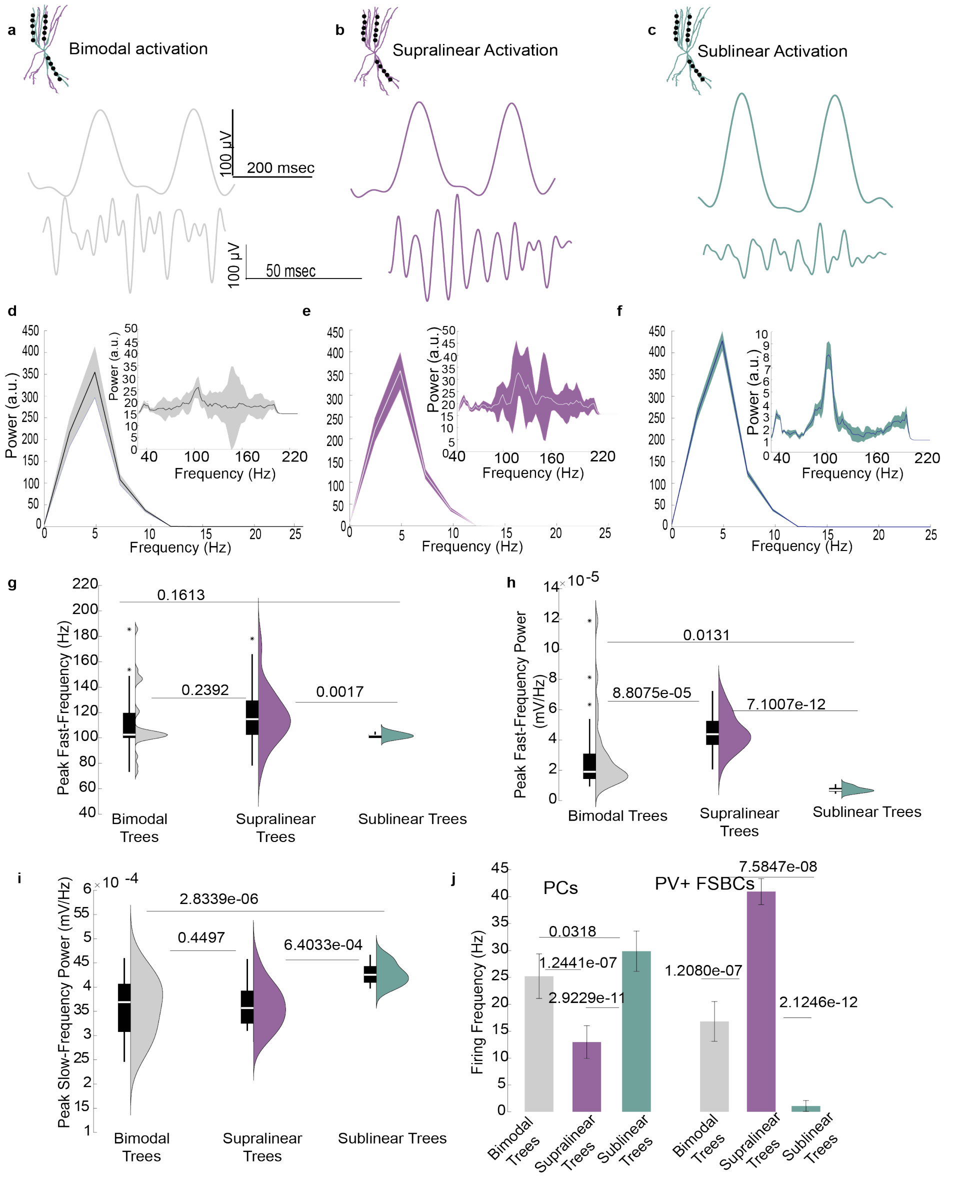

Figure S5. Impact of Clustered Synaptic Activation on LFP and E/I Balance in Bimodal, Supralinear, and Sublinear PV+ FSBCs Dendritic Configurations. a. Activation of PV+ FSBCs with bimodal nonlinear dendrites (control configuration), showing representative LFP traces for slow (3-10 Hz) and fast (30-200 Hz) frequencies. Synapses are clustered in a few randomly chosen supralinear or sublinear branches. **b**. Activation of Pv+ FSBCs with purely supralinear dendritic trees, showing LFP traces under clustered synaptic activation. **c**. Activation of PV+ FSBCs with purely sublinear dendritic trees, similar to conditions in a and b, illustrating the effect of clustered synaptic activation on LFP traces. **d-f**. Power Spectrum Density (PSD) plots for LFPs under clustered synaptic conditions in PV+ FSBCs with bimodal (d), supralinear (e), and sublinear (f) dendritic configurations. **g-i**. Comparison of peak frequency (g) and peak power for the fast LFP component (30-200 Hz) (h), and peak power for the slow LFP component (i), across dendritic configurations. **j**. Firing activity of PC and PV+ FSBC populations in the microcircuit network from 30 random simulation trials. Clustering in supralinear PV+ FSBC dendritic trees decreases the E/I balance in the network compared

to bimodal (control) or sublinear trees. Statistical analyses for multigroup comparisons were conducted using the Kruskal-Wallis test followed by a post-hoc correction for multiple comparisons. Paired comparisons and p-values were calculated using the Mann-Whitney U test for data with unequal variance. *Data show mean and std values.*

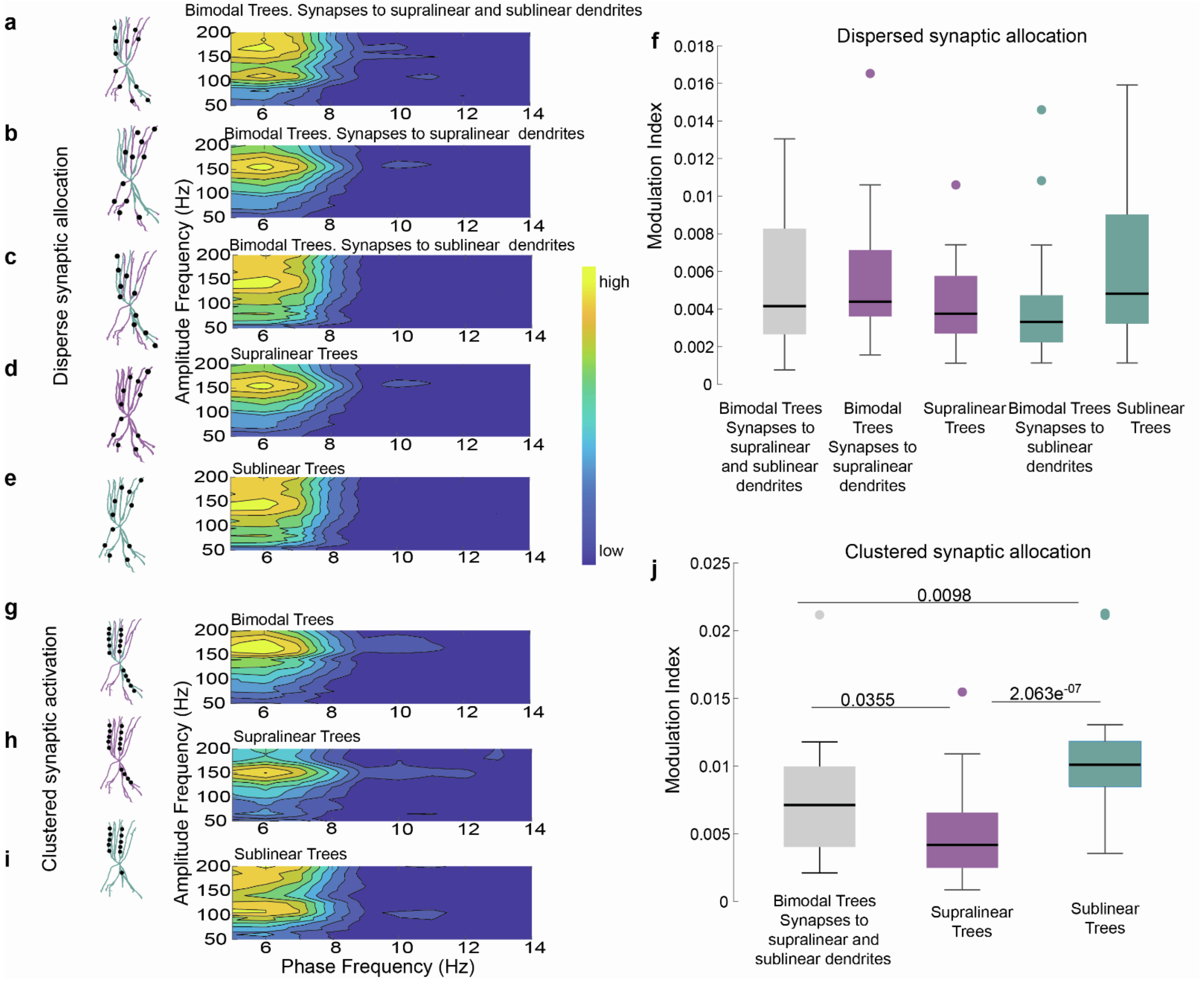

Figure S6. Slow-Fast Oscillation Coupling in the Microcircuit Model Under Various Dendritic and Synaptic Configurations in PV+ FSBCs. a-e. *Representative comodulograms illustrating the slow-fast coupling for the protocols detailed in Figures 2 and S3. These visualizations provide insights into the phase-amplitude coupling dynamics under different synaptic and dendritic configurations. Data from 30 random simulation trials are represented.* ***f.*** *Coupling analysis shows that synaptic distribution in a dispersed configuration across bimodal, purely supralinear, or purely sublinear PV+ FSBC dendritic trees does not affect slow-fast oscillation coupling.* ***g-i.*** *Similar to a-e but showcasing comodulograms for a clustered synaptic arrangement (detailed in Figure S4), using the same number of synapses as in the dispersed experiments.* ***j.*** *MI indicates that slow-fast oscillation coupling decreases when synapses are clustered in purely sublinear dendritic trees compared to other*

configurations. Multigroup comparisons were performed using the Kruskal-Wallis test followed by a post-hoc correction for multiple comparisons for multi-group data with unequal variance. This statistical approach was chosen to accommodate the diversity in the data from 30 random simulation trials. Data show mean and std values.

Data S1: *Glossary, terminology related to the whole manuscript text*

Glossary

Fast-Spiking Basket Cells (FSBCs)

GABAergic inhibitory interneurons that primarily target the perisomatic region of pyramidal cells and other GABAergic neurons. Most FSBCs express parvalbumin (PV+) and are characterized by their ability to fire at high frequencies.

PV+ neuron

Parvalbumin-positive interneuron.

SST+ neuron

Somatostatin-positive interneuron.

VIP+ neuron

Vasoactive Intestinal Polypeptide-positive interneuron.

Bimodal Dendritic Trees

Dendritic trees in which different branches exhibit distinct nonlinear modes of excitatory synaptic integration: specifically, supralinear and sublinear. These integration modes coexist within the same neuron and are governed by differences in the morphological and/or active properties of individual dendritic branches.

EPSPs (Excitatory Postsynaptic Potential)

Depolarizing electrical signals generated in the postsynaptic neuron upon the activation of excitatory synaptic input.

Dendritic Spikes

Local, nonlinear depolarizations in dendrites that reflect supralinear summation of excitatory postsynaptic potentials (EPSPs). Often modeled as a sigmoidal function of integrated EPSPs.

Memory Encoding

The cognitive process by which new information is initially acquired and stored—commonly referred to as learning.[70]

Memory Consolidation

The process by which newly encoded information is stabilized and stored for long-term retrieval.

Memory Recall

The retrieval of previously encoded and consolidated information.

E/I Balance (Excitation/Inhibition Balance)

The ratio of excitatory to inhibitory activity within a local circuit or network. An increase in inhibition lowers the E/I balance, while a reduction in inhibition raises it.

Neuronal Oscillations

Rhythmic fluctuations in electrical activity within the brain, typically measured via local field potentials (LFPs), that are associated with various behavioral and cognitive states.

Table S1. Active properties of PV+ Fast Spiking Basket Cell (PV+ FSBC) Models

Ion channel (S/cm^2)	*Soma*	*Axon*	*Proximal dendrites*	*Distal Dendrites*
Na_v	0.396	1.296	0.018	0.014
H_v	0.00001	X	x	X
Kdr_v	0.0432	0.144	0.0009	0.009
$Kslow_v$	0.000725	X	x	X
Kct_v	0.0001	X	x	X
Kca_v	0.02	X	x	X
Ka_v (proximal type) [64]	0.0032	X	0.001	0.0009
Ka_v (distal type) [65]	x	X	x	0.00216
Cal_v	x	X	0.00003	0.00003
Can_v	x	X	0.00003	0.00003
Cat_v	x	X	0.0002	0.0002
Calcium buffering dynamics	Yes	No	Yes	Yes

**values as per* [33,64,66]

Table S2. Passive properties of PV+ FSBCs

	Soma	*Axon*	*Proximal dendrites*	*Distal Dendrites*
Leak conductance (g_pas)[67]	1.315e-4 S/cm^2	3.55e-6 S/cm^2	1.315e-4 S/cm^2	1.34e-5 S/cm^2
Resting Membrane Potential (e_pas)[67,68]	-68 mV	-68 mV	-68 mV	-68 mV
Membrane capacitance (cm)[67]	1.2 uf/cm^{-2}	1.2 uf/cm^{-2}	1.2 uf/cm^{-2}	1.2 uf/cm^{-2}
Axial Resistance (Ra)[67]	172 ohm/ cm	172 ohm/ cm	142 ohm/ cm	142 ohm/ cm

Table S3. Passive Parameters and Active Conductance Values of the Pyramidal Cell (PC) Model

Mechanism	**Soma**	**Proximal Apical Dendrite(n=1)**	**Distal Apical Dendrites (n=2)**	**Basal Dendrites (n=2)**
Leak conductance [S/cm2]	0,0002	0,0002	0,0002	0,0002
Na+ conductance [S/cm2]	0,0105	0,0084	0,0084	0,0084
Delayed rectifier K+ conductance [S/cm2]	0,00086	0,00086	0,00086	0,00086
Proximal A-type K+ conductance [S/cm2]	0,0075	0,015	-	0,0075
Distal A-type K+ conductance [S/cm2]	-	-	0,04875	-
M-type K+ conductance [S/cm2]	0.06	0.06	-	0.06
Ih conductance [S/cm2]	0.00005	0.0001	-	0.00005
L-type Ca2+ conductance [S/cm2]	0.0007	0.00003	-	0.00003
R-type Ca2+ conductance [S/cm2]	0.0003	0.00003	-	-

T-type Ca2+ conductance [S/cm2]	0.00005	0.0001	-	0.0001
Ca2+ -dependent sAHP K+ conductance [S/cm2]	0.0015	0.001	-	0.0005
Ca2+-dependent mAHP K+ conductance [S/cm2]	0,9	0,03	-	0,08
Membrane capacitance Cm [µF/cm2]	1	1	1	1
Membrane resistance Rm [Ohm cm2]	6000	6000	6000	6000
Axial resistance Ra [Ohm cm]	150	150	150	150

*values as per [2, 33,62,67]

Table S4. Electrophysiological properties of the PCs and PV+ FSBCs models

	Value
Rheobase (pA)	200 (PC) / 150 +/- 10 (PV+ FSBC)
Input Resistance (MΩ)	142 (PC) / 97+/- 30 (PV+ FSBC)
Spike Threshold (mV)	-43,11 (PC) / -37 +/-1 (PV+ FSBC)
Spike Overshoot (mV)	30 (PC) / 48 +/- 1 (PV+ FSBC)
Resting Membrane Potential (mV)	-70 (PC) / -68 (PV+ FSBC)

Table S5. Synaptic conductance weight values of the PC and PV+ FSBC models.

Synapse Type	Pyramidal model	PV+ FSBC model
INPUT AMPA	0,00272	-
INPUT NMDA	0,0053	0.00032
INPUT CA++ PERMEABLE (CP)-AMPA	-	0.00075
AMPA	0,0017	-
CP-AMPA	-	0,0034

NMDA	0,0051	0.00144
GABA	0,0068	0,007
Autapse GABA	-	0,007

*values based on[29,33,56,57,59,63,69]
